# Current Progress of RNA Aptamer-Based Therapeutics

**DOI:** 10.3389/fgene.2012.00234

**Published:** 2012-11-02

**Authors:** Jiehua Zhou, Maggie L. Bobbin, John C. Burnett, John J. Rossi

**Affiliations:** ^1^Division of Molecular and Cellular Biology, Beckman Research Institute of City of HopeDuarte, CA, USA; ^2^Irell and Manella Graduate School of Biological Sciences, Beckman Research Institute of City of HopeDuarte, CA, USA

**Keywords:** RNA aptamers, systematic evolution of ligands by exponential enrichment, RNA interference, small interfering RNA, targeted delivery

## Abstract

Aptamers are single-stranded nucleic acids that specifically recognize and bind tightly to their cognate targets due to their stable three-dimensional structure. Nucleic acid aptamers have been developed for various applications, including diagnostics, molecular imaging, biomarker discovery, target validation, therapeutics, and drug delivery. Due to their high specificity and binding affinity, aptamers directly block or interrupt the functions of target proteins making them promising therapeutic agents for the treatment of human maladies. Additionally, aptamers that bind to cell surface proteins are well suited for the targeted delivery of other therapeutics, such as conjugated small interfering RNAs (siRNA) that induce RNA interference (RNAi). Thus, aptamer-siRNA chimeras may offer dual-functions, in which the aptamer inhibits a receptor function, while the siRNA internalizes into the cell to target a specific mRNA. This review focuses on the current progress and therapeutic potential of RNA aptamers, including the use of cell-internalizing aptamers as cell-type specific delivery vehicles for targeted RNAi. In particular, we discuss emerging aptamer-based therapeutics that provide unique clinical opportunities for the treatment various cancers and neurological diseases.

## Introduction

Aptamers are oligonucleotides selected *in vitro* to bind target molecules with high affinity and specificity due to their stable three-dimensional shapes. This high affinity is maintained *in vivo*, thereby enabling the aptamer to bind a specific receptor or ligand on the surface of the target cell. Hundreds of aptamers have been identified for specific functions and molecular targets, providing an unlimited number of possibilities for future therapeutics (Keefe et al., 2010).

**Figure 1:**
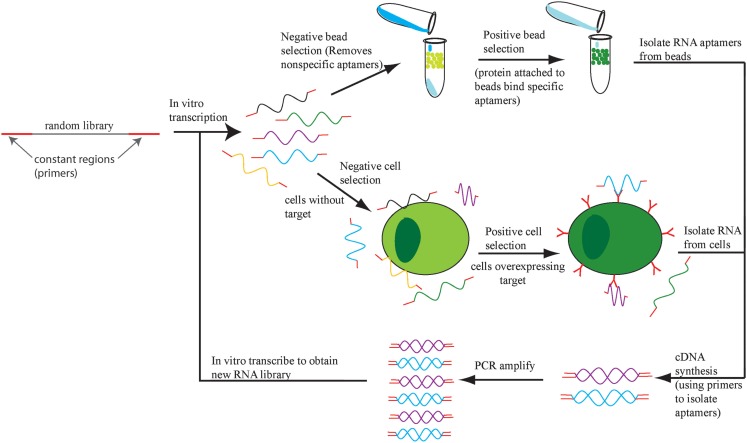
**The SELEX process**. An initial DNA library is transcribed into RNA and the aptamers that bind to cells or beads with no target protein are eliminated. The remaining aptamers are applied to cells or beads with target protein and the bound aptamers are retrieved, amplified, and the SELEX process repeats.

Aptamers are selected in a process called systematic evolution of ligands by exponential enrichment (SELEX; Ellington and Szostak, 1990; Tuerk and Gold, 1990). SELEX involves enriching for single-stranded RNA or DNA sequences based on retention of this sequence while it is bound to a target of interest (Figure 1). There are multiple variations of SELEX including cell-based SELEX and blind SELEX (Thiel et al., 2012). Cell-based SELEX involves selecting for aptamers that bind to an expressed protein and ensures that the aptamer targets the protein in its *in vivo* conformation. Blind SELEX selects an aptamer for an unknown target that is only present or more abundant in a diseased cell, such as an aptamer that binds to cancer cells but not healthy cells. Typically, the particular target is determined after the selection occurs. SELEX is an integral part of aptamer selection and is responsible for the broad range of targets for aptamer selection.

The function of aptamers is similar to that of current therapeutics including antibodies and small molecule therapy. Small molecules are about the same size or smaller than aptamers, and thus have comparable systemic clearance (Nimjee et al., 2005). However, aptamers offer a functional advantage over small molecule therapy, as aptamers are designed for cell specificity, thereby decreasing the potential off-target effects and toxicity of small molecule therapy. Aptamers are also similar to antibodies in that they tightly bind targets in a specific manner via structural elements and effect downstream pathways of the target molecule (Keefe et al., 2010). However, aptamers offer important advantages over their protein counterparts. The aptamer selection process is generally faster than monoclonal antibody selection and allows optimization of binding affinity by successive rounds of evolution and screening. While antibodies often elicit strong immune responses resulting in a loss of efficacy, immunogenicity is limited by aptamers due to chemical modifications on the aptamers. Due to their small size, aptamers have improved transport and tissue penetration than antibodies. Finally, in contrast to polyclonal antibodies, which must be selected *in vivo* resulting in a heterogeneous population, aptamers are synthesized *in vitro* as a homogenous population. As a result, production of aptamers offers greater quality control and lower expense compared to antibodies.

While RNA is notoriously prone to instability and degradation, chemical base modifications improve the stability of RNA aptamers (Mayer, 2009). Without chemical modification, the RNA aptamers will degrade and no longer bind or recognize targets. Modifications prevent RNase A mediated single-stranded RNA cleavage. Aptamers often have single-stranded loops that are responsible for target recognition. Therefore, the modifications keep hairpin loops and other single-stranded structures intact to maintain the binding affinity of the aptamer. Due to the increased resistance to RNases, aptamers are stable for over 2 h in human serum (Lorger et al., 2003). In addition to blocking RNase cleavage, modifications also increase the thermodynamic stability of aptamers. An increase in stability enhances the binding affinity of the aptamer to its target by ensuring the aptamer retains conformation. Incorporation of modified bases occurs by replacing pyrimidines with modified pyrimidines during *in vitro* transcription. Modifications include addition of fluorine (Fluoropyrimidine or F-Py), *O*-methyl (OMe) or amino group to the 2′ position of deoxyribose, which makes DNA bases appear as RNA bases for enhanced stability (Kawasaki et al., 1993).

## Aptamers as Therapeutics

Aptamers have shown early promise as therapeutics, as dozens of aptamers are in pre-clinical studies and at least nine have reached clinical testing (Ni et al., 2011a). Therapeutic strategies typically involve binding of the aptamer directly to a therapeutic target to modulate downstream signaling. Aptamer-based therapeutics for cancer include those that target adherence factors, immune system modulators, receptor tyrosine kinases (RTKs), and modulators of cell growth. Using the information gained from developing therapeutic aptamers for cancer, several aptamers have been developed to treat or identify neurological diseases. These include multiple sclerosis (MS), stroke, Alzheimer’s disease (AD), Parkinson’s disease (PD), variant Creutzfeldt–Jakob’s disease (vCJD), and neuronal cell death. In addition to therapeutic value as single agents, aptamers also provide a new strategy for the delivery of oligonucleotides and small molecules in a cell-specific manner. Aptamers are amenable to conjugation with other therapeutic nucleic acids or small molecules by covalent or non-covalent linkage. Indeed, this property has been utilized for a variety of chimeric molecules that contain an aptamer-conjugated with a small interfering RNA (siRNA) or short hairpin RNA (shRNA) that induce targeted RNA interference (RNAi). Effective knockdown is then observed upon treatment. To enhance the delivery of aptamer-siRNA chimeras, additional nanocarriers may be added to the complexes. The delivery and therapeutic utility of aptamers will be discussed in this article for *in vitro* and *in vivo* applications. The use of aptamers for the treatment of cancer and neurological diseases will be discussed in this section.

### Cancer

Therapeutic aptamers that engage with a specific target molecule on the surface of the cell can modulate downstream signaling pathways. Aptamers typically inhibit such signaling by preventing structural changes in the target molecule, inhibiting dimerization to associated molecules, or phosphorylation of downstream proteins (Esposito et al., 2011; Roth et al., 2012). Due to these and other properties, treatment of cancer is one of the leading uses for aptamers. Learning from research on the development of aptamers for cancer treatment, several groups are working on developing aptamers for the identification and treatment of neurological diseases.

Aptamers that are designed to distinguish between healthy and tumorigenic cells are especially useful for the detection and treatment of cancer. Cancer-targeting aptamers can be used for the selective treatment and elimination of only tumorigenic cells. Such specificity is needed for cancer treatment, as many current chemotherapeutics present side effects such as massive amounts of cell death and depletion of immune cells to fight infection (Bunka and Stockley, 2006; Burnett and Rossi, 2012). Hence, aptamer-mediated therapies are designed to minimize these adverse effects by not targeting non-tumorigenic cells and helping to maintain homeostasis in the patient. These aptamers treat various aspects of tumorigenesis, which will be discussed below (Table 1).

**Table 1:** **Therapeutic RNA aptamers for treatment of cancer**.

Aptamer target	Selection method	Model	Reference
E-selectin	Recombinant protein followed by cell-based SELEX	Cell culture	Mann et al. (2010, 2011)
Siacyl Lewis X	Siacyl Lewis X conjugated to agarose beads	Cell culture	Jeong et al. (2001)
CTLA-4	CTLA-4 antibody protein SELEX	Mouse model	Santulli-Marotto et al. (2003)
IL-4Rα	Magnetic epoxy beads attached to protein	Mouse model	Roth et al. (2012)
NOX-A12	Protein SELEX	Phase II clinical trial	Darisipudi et al. (2011)
HER3	Protein SELEX (rounds 1–8), isolation of band from gel shift (rounds 9–15)	Cell culture	Chen et al. (2003)
RET	Cell-based SELEX	Cell culture	Cerchia et al. (2005)
EGFR	Cell-based or protein-based (respectively)	Cell culture	Esposito et al. (2011), Li et al. (2011)
VEGF	Protein bound to beads	Mice-tumors FDA approved for human AMD	Bell et al. (1999), Huang et al. (2001)
Alpha-fetoprotein (AFP)	Protein bound to beads	Cell culture	Lee and Lee (2012)

### Adhesion

Metastasis may be prevented with aptamers that inhibit the formation of tumors at secondary sites. Several genes in the selectin family are highly overexpressed on cancer cells and promote cancer cell adherence to endothelial cells to form tumors at secondary sites (Nimjee et al., 2005). RNA aptamers to E-selectin and X, a carbohydrate responsible for adhering leukocytes to endothelial cells by interacting with selectins, have been selected for to inhibit cancer cell adherence (Jeong et al., 2001; Nimjee et al., 2005; Mann et al., 2011). The E-selectin aptamer blocks the interaction of E-selectin with ligands expressed by endothelial cells and decreases the adherence of cancer cells to endothelial cells (Mann et al., 2010, 2011) _ENREF_9. Sialyl-Lewis X aptamer works in a similar manner, by preventing the adherence of cancer cells to the endothelium. Sialyl-Lewis X is the preferred ligand for the selectins during attachment, so this aptamer prevents adherence to both P-selectin and E-selectin. Both aptamers prevent adhesion in cell culture thus far, but have not been tested *in vivo*.

#### Immune system manipulation

Modulation of the immune system is another important hallmark of tumorigenesis. Tumor cells inactivate the immune system to prevent tumor clearance. Aptamer inhibition of cytotoxic T-cell antigen 4 (CTLA-4) is one approach to prevent immune system inactivation and promote tumor elimination. CTLA-4 acts as an “off” switch for T-cell proliferation and therefore attenuates T-cell mediated immune responses (Santulli-Marotto et al., 2003). Anti-CTLA-4 aptamer promotes T-cell expansion and anti-tumor immunity in mice, as well as increasing the susceptibility of weakly immunogenic tumors to chemotherapy. It also can be used a tumor vaccine adjuvant by concurrent delivery of tumor antigen with aptamer to dendritic cells. As with antibodies, this aptamer can be used in a tetrameric form to enhance anti-tumor effects.

Another approach for enforcing the immune system against tumors is to inhibit the activity of tumor-associated macrophages (TAMs) and myeloid derived suppressor cells (MDSCs). Through activation of the IL-4Rα signaling pathway, these cells increase angiogenesis, promote tumor cell invasion, and inhibit anti-tumor immunotherapy. The IL-4Rα signaling cascade results in cell growth mediated by the activation of STAT1 and STAT6, which induces anti-apoptotic activity (Roth et al., 2012). Anti-IL-4Rα aptamer binding leads to depletion of MDSCs and TAMs, which inhibited tumor progression and size in mice.

Noxxon Pharma AG has developed an l-RNA (Spiegelmer) aptamer (NOX-A12) that binds to the chemokine (C-X-C motif) ligand 12 (CXCL12; Darisipudi et al., 2011), a ligand involved in tumor metastasis, cell homing, and angiogenesis (Keefe et al., 2010). NOX-A12 is a 45-nt l-RNA aptamer with a 3′-terminal PEG linkage (Sayyed et al., 2009). This therapeutic has been tested in two Phase I clinical trials to assess its safety and tolerability (NCT00976378 and NCT01194934). Currently, two Phase II clinical trials are scheduled for NOX-A12 for the treatment of lymphoma and multiple myeloma (NCT01521533 and NCT01486797).

#### Receptor tyrosine kinase

In addition to preventing metastasis, inhibition of tumor growth is important for the treatment of cancer. RTKs are cell surface receptors that are responsible for the regulation of key cell processes. Unregulated RTKs are often associated with tumor growth. Current clinically approved antibody therapies successfully target RTKs (Scott et al., 2012). Cetuximab targets endothelial growth factor receptor (EGFR), and Bevacizumab targets vascular endothelial growth factor (VEGF). Four aptamers have also been developed that prevent the growth of tumors by inhibiting RTKs: anti-HER3, anti-RET, anti-EGFR, and anti-VEGF (Huang et al., 2001; Chen et al., 2003; Cerchia et al., 2005; Esposito et al., 2011).

The RTK HER3 is responsible for increased drug resistance in HER2+ breast cancer (Chen et al., 2003). An aptamer-targeting HER3 inhibits stimulation of cell growth by the growth factor heregulin and prevents signaling through the HER3 receptor. Thus far, this aptamer has been tested *in vitro* and inhibits heregulin-induced proliferation of MCF7 cells. Since this aptamer is specific for HER3 signaling, it may therapeutically provide a safe alternative to currently used anti-cancer drugs such as tamoxifen or trastuzumab (Davies and Hiscox, 2011).

Anti-RET aptamer exhibits *anti-*cancer activity in cell culture by binding to the human receptor tyrosine kinase, RET (Cerchia et al., 2005). Mutated RET is an oncogene that upon dimerization causes thyroid carcinomas to develop by phosphorylating transcription factors involved in cell growth such as ERK, NF-κB, and AKT. Inhibition of RET dimerization prevents the phosphorylation of transcription factors involved in the proliferation of tumor cells. *In vivo* mouse studies of this aptamer are necessary to further demonstrate its efficacy and assess potential off-target effects.

Tumor cells over expressing the RTK EGFR are associated with poor prognosis due to phosphorylation-induced activation of AKT, STAT, and ERK pathways and to tumor cell proliferation, resistance to apoptosis, and metastasis (Esposito et al., 2011; Li et al., 2011). Anti-EGFR aptamers bind to EGFR monomers and heterodimers composed of EGFR and an additional RTK on the cell surface in cell culture (Li et al., 2011). This inhibits the activation of the downstream transcription factors that lead to increased tumorigenesis (Esposito et al., 2011). These aptamers also inhibit tumor growth in mice and induce apoptosis of tumor cells resistant to current antibody and chemical based anti-EGFR treatment, likely due to the aptamers small size and the inhibition of multiple forms of EGFR present on cell surfaces (Esposito et al., 2011).

Vascular endothelial growth factor is the ligand for VEGFA and VEGFB, both of which are RTKs that cause increased vascularization of blood vessels. An aptamer against VEGF has been used to treat pediatric Wilm’s tumors in mice by reducing tumor vascularization (Huang et al., 2001). Similar to VEGF antibody, the aptamer suppressed over 80% of tumor growth after 6 weeks, however, the aptamer is smaller and therefore may cause less off-target effects and toxicity compared to an antibody. This is critical for pediatric patient because off-target effects may prevent the growth of their developing blood vessels. VEGF165 is the epitope targeted by this aptamer, which is the same epitope that is targeted by an aptamer clinically approved for age-related macular degeneration (AMD) called Pegaptanib (Vinores, 2006; Tolentino, 2011). Pegaptanib effectively treats AMD by halting blood vessel growth in the eye, preventing blindness.

#### Cell expansion

Hepatocellular carcinoma is often associated with production of alpha-fetoprotein (AFP). AFP is associated with liver cancer development due to cell growth and differentiation of hepatic cells. Interaction of AFP with an AFP receptor results in the stimulation of oncogenic mRNA production. As a result, an aptamer has been developed that binds to AFP, reduces cell viability, and reduces downstream oncogene expression in cell culture (Lee and Lee, 2012). This aptamer may eventually be used for the treatment and analysis of hepatocellular carcinoma.

### Neurological disease

Diseases of the nervous system are difficult to identify and treat because it is difficult to access the nervous system (Lindvall and Kokaia, 2006). Once identified by brain biopsy or some other method, treatment options are scarce. Aptamers provide viable options for treatment: small size allows for deep tissue penetration and crossing of the blood brain barrier, high affinity reduces the amount of aptamer needed for treatment and reduces the likelihood of side effects, and easy regulatory mechanisms adds a fail-safe to the treatment (de Franciscis et al., 2009). Many of the therapies discussed in this section are similar to strategies used for the treatment of cancer. Regulation of the immune system is often important for treating neurological disorders, while mobilization of the immune system is critical for cancer treatment. Aptamers have been developed for treatment of several conditions targeting MS, stroke, AD, prions, and other proteins (Table 2).

**Table 2:** **Therapeutic RNA aptamers for treatment of neurological disease**.

Aptamer	Selection method	Application	Model	Reference
IL-17	Protein bound to beads	MS	Mouse	Ishiguro et al. (2011)
Midkine	Protein bound to beads	MS	Mouse	Muramatsu (2011)
Factor IXa	Protein bound to nitrocellulose	Stroke	Mouse-stroke Humans-phase II clinical trial for acute coronary syndrome	Rusconi et al. (2002)
BACE1	Protein bound to sepharose beads	Alzheimer’s	*In vitro*	Rentmeister et al. (2006)
Aβ	Protein bound to sepharose beads	Alzheimer’s	*In vitro*	Ylera et al. (2002)
Prion protein (PrP)	Protein bound to nitrocellulose	vCJD	*In vitro*	Murakami et al. (2008)
AMPA	Membrane bound protein	Stroke, Parkinson’s, treat neuronal cell death	*In vitro*	Park et al. (2011)
Nogo-66	Protein bound cellulose	Promote myelin sheath regeneration, neuronal growth	Cell culture	Wang et al. (2010)

#### Multiple sclerosis

Multiple sclerosis is an incurable disease caused by inflammation of the CNS identified by leukocyte infiltration in the CNS. Treatment options available merely modify the disease progression or treat the symptoms present. Experimental autoimmune encephalitis (EAE) is an animal disease that closely resembles MS and serves as a mouse model to study MS disease progression and therapies. Since the cause of MS has not been determined, aptamers play a role in determining the biology of EAE and MS in addition to providing new treatment options. Two such aptamers are the IL-17 aptamer and the midkine aptamer. IL-17 aptamer inhibits the proinflammatory activity of IL-17, reducing the neurological symptoms observed in the EAE mouse model (Ishiguro et al., 2011). Another aptamer against midkine may be useful for the treatment of MS because the aptamer reduced inflammation observed in mouse EAE (Wang et al., 2008b; Muramatsu, 2011). Midkine promotes neuronal and tumor cell growth in addition to inhibiting regulatory T-cell expansion. MS is characterized by a lack of T-cell regulation, so midkine repression may reduce the neuroinflammation by inducing regulatory T-cells to control the immune system.

#### Stroke

Ischemic strokes occur when blood flow to the brain ceases usually because of a blood clot in the brain or other part of blood vasculature (Blake et al., 2011). Inflammation and lack of blood flow from the ischemic stroke often lead to permanent brain damage. Tissue plasminogen activator (TPA) is the only approved treatment for stroke that acts by dissolving blood clots allowing normal blood flow to resume, however, TPA is recommended to be given within 4.5 h of stroke onset (Demers et al., 2012). This restricts the number of patients able to receive TPA, which is further restricted because TPA treatment also can cause hemorrhaging. Due to this complication, there is a need for better therapy with a mechanism for inactivating the therapeutic to prevent hemorrhaging. An aptamer candidate with similar activity to TPA is the Factor IXa aptamer, RB006, which Regado Biosciences currently has Phase II clinical trials for use in acute coronary syndrome (NCT00932100 and NCT00113997; Blake et al., 2011; Burnett and Rossi, 2012). Factor IXa is a clotting factor, so inhibition by an aptamer dissolves blood clots in addition to reducing inflammation and improving neurological function in mice. To prevent hemorrhaging, a mechanism for inactivating the aptamer is available using aptamer antidote RB007. This aptamer antidote binds and inactivates the Factor IXa aptamer, therefore allowing Factor IXa levels to return to normal. As a stroke treatment, this presents a safe, regulated alternative to TPA.

#### Alzheimer’s disease

While the particular causes AD remain unknown, the deposition of β-amyloid (Aβ) protein seems to disrupt the functional connectivity of neurons in healthy areas of the brain (Bero et al., 2012). Aβ also causes synuclein accumulation in the brain and PD (Masliah et al., 2001). Diagnosis occurs by a brain biopsy for AD, and no treatment for AD or PD is available mostly due to a lack of information about the diseases. Since few targets exist for AD or PD, a biomarker assay using SOMAmers has been developed (Baird et al., 2012). SOMAmers are aptamers with different functional groups on uracil residues that enhance the stability and functionality of the aptamer. This assay was developed to test age-related differences in proteins found in the cerebrospinal fluid. The authors identified over 200 biomarkers of aging which may be used to help diagnose and eventually treat AD or PD.

An aptamer that targets the β-secretase (BACE1) protein is designed to inhibit the formation of Aβ for treatment of AD (Rentmeister et al., 2006). Aβ is created by the cleavage of β-amyloid precursor protein by BACE1. The anti-BACE1 aptamer may help to elucidate the cause of AD and provide new treatment options, but still must undergo further testing in culture or animal models. Two different aptamers against Aβ present another possible therapeutic for the treatment of AD. The goal of the first aptamer was to select an aptamer that bound to the multimeric Aβ, but did not recognize monomeric Aβ (Ylera et al., 2002). This aptamer was successful for the marking of Aβ fibrils and in the future, may be used for the diagnosis or treatment of AD. A second aptamer was developed that also recognized Aβ fibrils and other amyloid proteins *in vitro* (Rahimi et al., 2009; Rahimi and Bitan, 2010). This aptamer does not recognize monomeric or oligomeric Aβ even though the aptamer was selected against multimeric Aβ. Nevertheless, this aptamer may serve as an improved diagnostic tool than current assays due to increased aptamer sensitivity for AD fibrils.

#### Variant Creutzfeldt–Jakob disease

Although distinct from classic Creutzfeldt–Jakob disease (CJD), vCJD is thought to be closely related to mad cow disease and is caused by deposition of an insoluble form of prion protein (PrP) in the brain (Murakami et al., 2008). It is currently unknown what causes the structural change from the soluble form to the insoluble, infectious form. PrP binds to nucleic acids making it difficult to obtain a functional aptamer, yet several aptamers have been developed (Proske et al., 2002; Rhie et al., 2003; Murakami et al., 2008). Two aptamers may serve as therapeutics for vCJD due to their recognition of insoluble PrP over soluble PrP *in vitro*. The aptamers also prevented the formation of insoluble, infectious PrP. A third aptamer recognizes bovine PrP and was developed to detect PrP in bovine brain homogenate with possible application in humans (Murakami et al., 2008). This aptamer may play a role in preventing PrP contaminated beef from reaching the food supply. The other two aptamers have the possibility for therapeutic use in humans and the prevention of PrP formation.

#### Broad effectors

Neuron cell death due to uptake of too much Ca^2+^ through the glutamate ion channel, α-amino-3-hydroxy-5-methyl-4-isoxazolepropionic acid (AMPA) receptor, is the cause of many neurological diseases (Dingledine et al., 1999; Huang et al., 2009; Park et al., 2011). AMPA receptor is responsible for fast synaptic transmission in the CNS and is composed of multiple subunits. Aptamer targets the closed conformation of the GluA2 subunit of the AMPA receptor, which may regulate Ca^2+^ flow, but has not been tested in cell culture. Aptamers to GluA2 have potential for therapeutic use in treating stroke, PD, and other diseases hallmarked by neuron death. These aptamers allow for tight control of the AMPA receptor and may be therapeutically valuable for many neurological diseases characterized by abnormal AMPA activity.

Another important process in reversing the effects of neurodegenerative disease is generating new neurons, which can be accomplished by the regeneration of myelin sheath that coats axons within the central and peripheral nervous systems (Wang et al., 2010). Inhibition neuron growth and regeneration occurs via Nogo-66 (NgR) receptor association with p75 leading to the activation of RhoA, an inhibitor of myelin sheath regeneration and axon elongation (Yamashita and Tohyama, 2003). *Anti*-NgR aptamer binds with high affinity to NgR, prevents the association of NgR with p75, and competes with agonists of NgR/p75 association (Wang et al., 2010). Growth of axons in neurite outgrowth assays also occurs upon treatment with aptamer.

## RNA Aptamer-Mediated Targeted Therapy

By binding and blocking enzyme activities or protein–protein interaction, aptamers alone show the promise as new class of therapeutic agents in the treatment human maladies (Nimjee et al., 2005; Bunka and Stockley, 2006; Keefe et al., 2010). In addition, cell-specific aptamers that actively target a distinct cell population or tissue also show the potential in therapeutic targeting or delivery (Syed and Pervaiz, 2010; Zhou and Rossi, 2010, 2011). For instance, cell-specific aptamers may be functionalized with other therapeutic agents or delivery vehicles, such as siRNA, shRNA, microRNA (miRNA), toxins, enzymes, chemotherapy agents, photodynamic molecules, radionuclides, and nanocarriers. Such design for the aptamer-mediated targeted therapy may enhance therapeutic index as well as attenuate the overall toxicity of the drugs (Dua et al., 2011; Tan et al., 2011).

In present, an increasing number of cell-specific RNA aptamers have been successfully adopted for targeting delivery of various cargoes *in vitro* as well as *in vivo*. The cell-specific RNA aptamers used as delivery ligands or vehicles for various cargoes in cancer therapy are summarized in Table 3. Different strategies have been employed to functionalize RNA aptamer to the cargoes or carriers. For example, due to the nucleic acid nature of the RNAi therapeutics, siRNA, or shRNA molecules can be directly fused to a RNA aptamer backbone to achieve a chimeric RNA for the delivery purpose (Zhou and Rossi, 2010). Alternatively, aptamer and therapeutics also can be non-covalently assembled via an adapter linkage. In addition, aptamers can be chemically synthesized with specific functional groups on 5′- or 3′-termini for conjugation with other therapeutics or drug-loaded nanocarriers. The following sections mainly discuss current promising pre-clinical studies where cell-specific RNA aptamers have been utilized for targeted RNAi. Several representative examples of aptamer-mediated RNAi are highlighted below.

**Table 3:** **Cell-type specific RNA aptamers for targeted cancer therapy**.

Target name	Specification of RNA aptamer	Target and technique for RNA aptamers selection	Representatives of RNA aptamer-mediated targeted delivery systems *in vitro* and/or *in vivo*	Reference
Prostate-specific membrane antigen (PSMA)	2′-F-Py modification (A10, A9 aptamer and truncated of A10 aptamer: A10-3 and A10-3.2)	Purified fusion protein containing a modified extracellular form of PSMA *via* magnetic beads-based SELEX	(1) siRNA delivery: non-covalent A9 aptamer: lamin A/C or GAPDH siRNA conjugates (PSMA-positive LNCaP cells)	Chu et al. (2006a)
		(2) siRNA delivery: covalent A10 or A10-3.2 aptamer-PLK1, BCL2, or NMD siRNA chimeras (PSMA-positive LNCaP cells; B16/F10 cells; athymic nude mice; C57BL/6 mice)	McNamara et al. (2006), Dassie et al. (2009), Pastor et al. (2010)
			(3) siRNA delivery: bivalent A10-3 aptamer – EEF2 siRNA chimeras (PSMA-positive LNCaP cells)	Wullner et al. (2008)
			(4) siRNA-nanoparticles delivery: aptamer – S-S – siRNA chimeras – cationic PEI-coated QD NP conjugates (PSMA-positive C4-2B cells)	Bagalkot and Gao (2011)
			(5) shRNA delivery: bivalent A10-3 aptamer – EEF2 shRNA chimeras; covalent A10-3 aptamer – DNAPK shRNA chimeras (PSMA-positive LNCaP cells; xenografts; human prostate tissues)	Wullner et al. (2008), Ni et al. (2011b)
			(6) shRNA-nanoparticles delivery: Bcl-xL shRNA-loaded covalent aptamer-Dox-PEI conjugates (PSMA-positive LNCaP cells); AR shRNA-loaded A10 aptamer – polymer conjugates (xenograft models with different prostate cancer cell lines: 22RV1, LAPC-4, and LNCaP)	Kim et al. (2010)
			(7) miRNA-nanoparticles delivery: miRNA (miR-15a and MiR-16-1) loaded covalent A10-3.2 aptamer – PEG – PAMAM dendrimer conjugates (PSMA-positive LNCaP cells)	Wu et al. (2011)
			(8) Chemo-immune agent – nanoparticles delivery: A9 aptamer – immune-stimulating agent CpG – Doxorubicine – PAMAM dendrimer conjugates (PSMA-positive LNCaP cells; xenograft tumor model)	Lee et al. (2011)
			(9) Protein delivery: covalent aptamer: gelonin (toxin) conjugates (PSMA-positive LNCaP cells)	Chu et al. (2006b)
			(10) Chemotherapeutic agents encapsulated nanoparticles delivery: aptamer – Dextran, Docetaxel, Pt(IV) or Doxorubicin encapsulated NPs conjugates (PSMA-positive LNCaP cells; BALA/c nude mice)	Farokhzad (2004, 2006), Dhar et al. (2008), Cheng et al. (2007), Wang et al. (2008a)
			(11) Anthracycline delivery: aptamer-Doxorubicin physical conjugates (PSMA-positive LNCaP cells)	Bagalkot et al. (2006), Zhang et al. (2007), Gu et al. (2008), Min et al. (2011)
Cluster of differentiation 4 (CD4)	2′-F-Py-modification	Recombinant soluble CD4 antigen *via* sepharose beads-based SELEX	siRNA-pRNA nanostructure delivery: non-covalent phi29 pRNA/aptamer – pRNA/siRNA dimer or trimer (CD4 overexpressing T-cells)	Guo et al. (2005), Khaled et al. (2005)
Human receptor activator of NFêB (RANK)	G-quartet structure; specifically binds to CD30 (K_d_ 0.11 nM)	Recombinant receptor activator of NFêB (RANK) protein *via* TALON metal affinity resin-based SELEX	siRNA-nanoparticles delivery: non-covalent aptamer and ALK siRNA encapsulated PEI-NPs (CD30-expressing Karpas 299 cells)	Mori et al. (2004), Zhang et al. (2009) Zhao et al. (2011)
Epidermal growth factor receptor 2 (HER2)	2′-F-Py modification	HER2-expressing N202.1 A cells *via* Cell-based SELEX	siRNA delivery: covalent HER2 aptamer-Bcl2 siRNA chimeras (HER2-positive murine mammary tumor cell lines 85819)	Thiel et al. (2012)
Tenasin-C (TN-C)	2′-F-Py and 2′-OMe purine substitutions	Purified TN-C protein *via* 96-well lumino plate-based SELEX; TN-C-expressing U251 glioblastoma cells *via* Cell-based SELEX; or crossover SELEX	Radionuclide agent delivery: covalent aptamer – ^99m^Tc conjugates (Glioblastoma tumor cells; nude mice)	Hicke et al. (2001, 2006)
Epidermal growth factor receptor (EGFR)	2′-F-Py modification	Purified extracellular domain of human EGFR protein *via* cellulose filter-based SELEX	Gold nanoparticles delivery: non-covalent aptamer – DNA – linker – gold NP conjugates (A431 cells)	Li et al. (2010)
Transferrin receptor (TfR)	5′-biotinylation (FB4 aptamer)	Recombinant extracellular domain of the mouse TfR *via* nitrocellulose filter binding and affinity spin column	Protein delivery: non-covalent biotinylated FB4 aptamer – streptavidin conjugates (LtK^−^ cells)	Chen et al. (2008)

*SELEX, systematic enrichment of ligands by exponential enrichment; EEF2, eukaryotic elongation factor 2; siRNA, small interfering RNA; shRNA, short hairpin RNA; PK, protein kinase; QD, quantum dot; PLK1, polo-like kinase 1; NMD, nonsense-mediated mRNA decay; Bcl2, B-cell lymphoma 2; PSMA, prostate-specific membrane antigen*.

### Aptamer-mediated siRNA/shRNA delivery

As mentioned above, aptamer-mediated specific recognition relies essentially on their unique 3D structure to their targets (Mayer, 2009). Other types non-coding RNA, such as siRNA or small hairpin (sh)RNA, function by specific sequence complementarity to target mRNA (Fire et al., 1998; Elbashir et al., 2001). However, the efficient and safe delivery of RNAi-based therapeutics into specific tissue or cell populations is still the principal challenge in the clinical development of RNAi (Whitehead et al., 2009; Higuchi et al., 2010; Davidson and McCray, 2011). By combining the two therapeutic non-coding nucleic acids into a single chimeric molecule, the resulting targeted RNAi therapeutics could further enhance therapeutic potency.

#### Non-covalent aptamer-siRNA conjugates

To date, the most established and best characterized cell-specific RNA aptamers for targeted therapy are the 2′-fluoro modified anti-PSMA aptamers with low nanomolar affinity binding constants (Lupold et al., 2002). PSMA, a transmembrane protein, is highly expressed in human prostate cancer and the vascular endothelium and is continually recycled from the plasma membrane (Liu et al., 1998; Tasch et al., 2001). The parental anti-PSMA aptamers (A9 and A10) and its truncated versions (A10-3 and A10-3.2) have been successfully employed for specifically delivery of siRNAs or shRNAs *in vitro* and *in vivo* animal model (Table 3).

**Figure 2:**
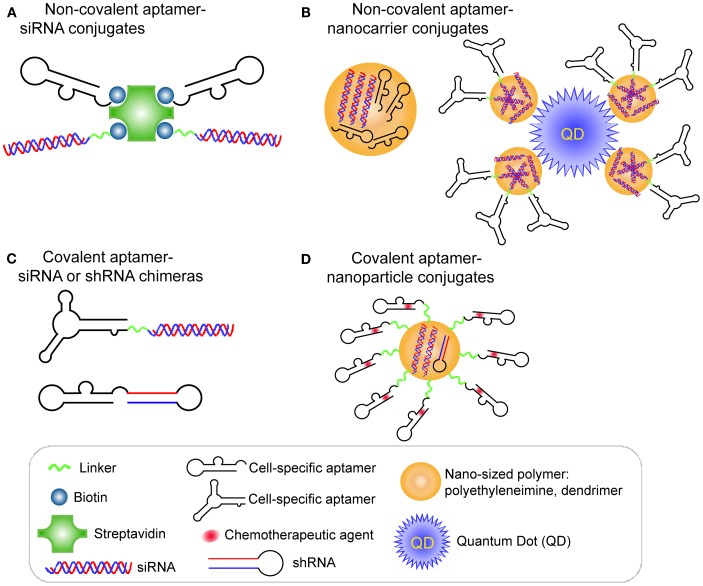
**RNA aptamer-mediated targeted RNAi delivery in cancer therapy**. **(A)** Schematic of non-covalent aptamer-siRNA conjugates. Cell-specific aptamers and 27-mer Dicer substrate siRNA duplexes were chemically conjugated with a biotin group. Thus, two biotinylated DsiRNAs and two aptamers were non-covalently assembled *via* a Streptavidin platform. **(B)** Schematic of covalent aptamer-siRNA or shRNA chimeras. The 2′-Fluoro modified aptamer and siRNA sense strand were co-transcribed, followed by annealing of the complementary siRNA antisense strand to complete the chimeric molecule. Or the aptamer and shRNA strand were co-transcribed to obtain the 2′-Fluoro modified chimeric RNA. **(C)** Schematic of non-covalent aptamer-nanocarrier conjugates. A nanocomplex was formulated by incorporating siRNAs and aptamers onto polyethyleneimine (PEI)-citrate nanocarriers *via* electrostatic interaction. Or the siRNAs with SH group were absorbed on PEI-coated quantum dot nanoparticles *via* non-covalent electrostatic interaction. And then aptamers with a single thiol group were coupled to siRNAs *via* thiol-disulfide exchange reaction to form aptamer-siRNA chimeras.**(D)** Schematic of covalent of aptamer-nanoparticle conjugates. A 5′-NH_2_-modified aptamer was chemically synthesized and covalently decorated on the surface of a polymer composed of branched polyethyleneimine grafted with polyethylene glycol (PEI-PEG) for co-delivery of shRNA and chemotherapy agents.

A proof of concept study by Ellington et al. (Chu et al., 2006a) validated the aptamer-mediated target delivery technology using the A9 anti-PSMA aptamer. Two biotinylated A9 anti-PSMA aptamers and two biotinylated 27-mer Dicer substitute siRNAs (DsiRNAs) against lamin A/C or GAPDH were non-covalently assembled on a streptavidin bridge through biotin-streptavidin interaction (Figure 2A). When the resulting multivalent conjugates were incubated with PSMA-positive LNCaP cells or the control PC-3 cells, selective cellular uptake and specific RNAi activity was observed in LNCaP cells, suggesting targeting specificity mediated by aptamer-siRNA conjugates.

#### Covalent aptamer-siRNA or shRNA chimeras

A different strategy was used to develop a covalent aptamer-siRNA chimera using the A10 anti-PSMA aptamer (McNamara et al., 2006). In this approach, a 2′-fluoro modified PSMA aptamer was directly fused with the passenger strand of a 21-mer siRNA duplex, which subsequently was annealed with its complementary guide strand (Figure 2B). The resulting A10 aptamer-PLK1 or BCL2 siRNA chimeras induced specific silencing of target genes and cell death in prostate cancer cells expressing PSMA. Additionally, in an athymic mouse model the intratumoral injection of the aptamer-siRNA chimeras allowed effective gene down-regulation and tumor regression.

The anti-PSMA aptamer-siRNA has undergone further optimization to improve the *in vivo* therapeutic potency and circulating half-life of the anti-PSMA aptamer-siRNA chimera (Dassie et al., 2009). By rational design of the aptamer and siRNA, the same group optimized the previous A10 aptamer-PLK1 siRNA chimera for systemic administration by implementing three important features. First, the aptamer portion was truncated from 71 (A10 aptamer) to 39 nucleotides (A10-3.2) which still preserves original specificity and affinity to PSMA, thus allowing a large-scale solid-phase synthesis of the chimeras. Second, a 2-nt overhang was added at the 3′-end of guide strand of siRNA portion or the guide and passenger strands of siRNA portion were swapped to append to the aptamer portion to facilitates Dicer access, processing and loading, thus improving RNAi activity by about 100-fold. Third, the 5′-end of the passenger strand of siRNA portion was conjugated with a 20-kDa polyethylene glycol (PEG) linkage, which increases the molecular weight and improves its pharmacokinetic profile. Systemic administration of the optimized aptamer-siRNA chimera, termed swap-2′F-PEG chimera, in immune-compromised mice bearing PSMA-expressing prostate tumors yielded increased circulating half-life (from <35 min to >30 h) and bio-availability. Moreover, the swap-2′F-PEG chimera provided prolonged silencing and pronounced tumor regression at significantly lower dosage (5 × 0.25 nmol pre-injection) than an earlier version of the chimera lacking PEG (swap-2′F chimera), which reduces the risk of harmful side effects and the treatment costs.

Since the introduction of the simple covalent aptamer-siRNA chimera, several different groups have fused various siRNAs to the A10 anti-PSMA aptamer and its truncated versions for siRNA or shRNA delivery. Wullner et al. (2008) designed two bivalent anti-PSMA aptamer-EEF2 siRNA chimeras, in which the siRNA portion serviced either as a spacer joining the two A10-3 anti-PSMA aptamers or as a shRNA format to be tethered to the 3′ end of one of the aptamer dimers. The incubation with PSMA-positive prostate cancer cell resulted in promoting up to four times more cellular uptake of chimeras than their monovalent A10-3 aptamer-EEF2 shRNA chimera, therefore enhancing RNAi efficacy.

A recent study from Gilboa and his coworkers also successfully applied the anti-PSMA aptamer-siRNA chimera strategy to the field of cancer immunotherapy (Pastor et al., 2010). The A10 aptamer was employed to deliver anti-UpF2 and anti-Smg1 siRNAs to PSMA-expressing mouse tumor cells in culture. The systemic administration (intravenous) of anti-PSMA aptamer-siRNA chimeras also resulted in significant suppression of tumor growth in subcutaneous and metastatic tumor animal model. Additionally, upon high, long-term doses of the chimeras, most mice treated completely rejected the tumor cells, which suggested aptamer-mediated tumor-specific immunotherapy is a potential approach for eradicating cancer.

Currently, combination therapy for cancer using multiple treatments (such as radiation and chemotherapy) has shown apparent therapeutic benefits. However, escalated doses and non-targeting cell killing may increase unwanted side effect and injury to non-cancerous cells or tissues. Ionizing radiation (IR) causes multiple types of cellular injury, which can be sensitized by chemical or siRNA inhibition of DNA repair protein (Collis et al., 2003; Ohnishi et al., 2006; Chalmers et al., 2007). In a recent study, Lupold et al. described a prostate-targeted RNAi agent selectively sensitized PSMA-positive cells to IR therapy (Ni et al., 2011b). Through a high-throughput RNAi screen, several novel siRNAs were identified against DNA-activated protein kinase, catalytic polypeptide (DNAPK) from siRNA library. Using an established screening approach (McNamara et al., 2006), a PSMA aptamer A10-3–DNAPK shRNA chimera was generated (Figure 2B), which selectively reduced DNAPK gene expression in PCa cells, xenografts, and human prostate tissue (Ni et al., 2011b). Moreover, the aptamer-shRNA chimera functioned as a selective sensitizing agent, as it dramatically and specifically enhanced PSMA-positive tumor response to IR, therefore reducing non-specific injury of IR to health cells or tissues.

Most recently, an alternative cell-internalizing RNA aptamer that binds to the human epidermal growth factor receptor 2 (HER2) was developed for delivering therapeutic siRNA to HER2-expressing breast cancer cells (Thiel et al., 2012). HER2, one of members in the epidermal growth factor receptor (EGFR) family, is a key player in breast cancer (Sundaresan et al., 1999; Yarden, 2001; Moasser, 2007). Compared to the cancer lacking HER2 expression, HER2^+^ breast cancers is more aggressive and more likely to be resistant to therapy (Higa et al., 2010; Davies and Hiscox, 2011). In this study, the selected anti-HER2 aptamer was covalently fused with Bcl2 siRNA to specifically induced gene silencing in HER2^+^-cells, which sensitized these cells to chemotherapeutic cisplatin (Thiel et al., 2012).

### Aptamer-mediated siRNA/shRNA-loaded nanocarrier delivery

Compared with traditional therapeutics, nanocarrier-based therapeutics are capable of bearing various surface modifications (e.g., targeting ligands, PEG) and accommodate multiple drugs (e.g., RNAi therapeutics and small molecular drugs) simultaneously, thus altering drugs biodistribution, resistance, and pharmacokinetics (PK). Nanocarriers can be decorated with ligand-specific aptamers to achieve actively cell-type for delivery. Hence, aptamers can be used direct targeted delivery for a variety of nanoparticles, including chemically synthesized polymers, liposomes, Au-nanoparticles, quantum dots (QD), supraparamagnetic iron oxide nanoparticles (SPION), single wall carbon nanotubes (SWCNT; Levy-Nissenbaum et al., 2008; Thiel and Giangrande, 2009, 2010). Moreover, the large surface areas and the interior cavities of nanocarriers can provide excellent platforms for conjugating multiple aptamers and storing large quantities of various therapeutic molecules, thereby increasing loading capacity. Toward this end, RNAi-based therapeutics have been loaded into various aptamer-functionalized nanocarriers for targeted delivery of the siRNA (Burnett et al., 2011).

#### Non-covalent aptamer-nanocarrier conjugates

As mentioned before, the interior volumes of nanoparticles can be used to encapsulate large quantities of drug molecules. A nanocomplex was formulated by incorporating both an anaplastic lymphoma kinase (ALK) siRNA and an anti-CD30 RNA aptamer (Mori et al., 2004; Zhang et al., 2009) onto polyethyleneimine (PEI)-citrate nanocarriers *via* non-covalent interaction (Zhao et al., 2011; Figure 2C). CD30 is a cell membrane protein of the tumor necrosis factor receptor family that has been recognized as a unique biomarker on many lymphomas of diverse origin and as activation molecule on B- and T-cells (Duyster et al., 2001). After directly mixing individual siRNAs and aptamers with PEI to form an average ∼140 nm hydrodynamic diameter, the targeted nanocomplexes specifically silenced ALK gene expression and induced growth arrest and apoptosis in CD30-expressing anaplastic large cell lymphoma (ALCL) cells (Zhao et al., 2011).

After entry of the aptamer-siRNA nanocomplexes into the targeted cell, the therapeutic must escape from the endosome to confer the siRNA function. With this in mind, Gao et al. (Bagalkot and Gao, 2011) rationally designed a multifunctional nanoparticle (PEI-coated quantum dot) system that is equipped with a proton sponge effect to enhance endosomal escape (Figure 2C). Additionally, the nanocomplex displays a large surface area for high siRNA payload, exposed aptamer for specific targeting, and fluorescence for imaging and qualification. Compared with the conventional one-step incorporation of individual siRNA/shRNA and aptamers onto a nanoparticle with random orientations and conformations, this design requires a two-step process to graft aptamer-siRNA chimeras onto nanoparticles. In this design, the siRNAs with a thio-reactive terminal group are firstly adsorbed on PEI-coated QD nanoparticles *via* non-covalent electrostatic interaction to reduce nanoparticle surface charge, thus avoiding some non-specific electrostatic interactions between negatively charged aptamers and nanoparticles surface. Next, PSMA aptamers with a single thiol group are coupled to siRNAs *via* thiol-disulfide exchange reaction to form aptamer-siRNA chimeras. Because of the reduced positive charges on nanoparticles, their interaction with aptamers is weakened, thus helping to retain aptamer accessibility, flexibility, and binding affinity. The resulting PSMA aptamer-targeting nanoparticles displayed selective gene silencing and enabled 34% more silenced cells of the total cell population over non-targeted nanoparticle-siRNA complexes (Bagalkot and Gao, 2011).

Using a different strategy, Guo and coworkers fabricated an anti-CD4 aptamer into a multifunctional nanostructure for targeted delivery of siRNAs in a T-cell line expressing the human CD4 receptor (Guo et al., 2005; Khaled et al., 2005). CD4 is a glycoprotein expressed on the surface of certain subsets of T lymphocytes (Dalgleish et al., 1984; Hussey et al., 1988). The CD4 receptor can be endocytosed in T helper cells, internalizing itself and any cargo to which it is bound (Pelchen-Matthews et al., 1991). By using a soluble, recombinant CD4 antigen immobilized onto sepharose beads, Kraus et al. (1998) successfully identified several 2′-F-modified RNA aptamers, which blocked functional T-cell responses. The packaging RNAs (pRNAs), derived from bacteriophage phi29 small RNAs, contain a 5′/3′ helical domain and an intermolecular interaction domain. pRNA monomer can fold into a stable and unique secondary structure that serve as the building blocks to form various nanostructures (e.g., dimer, timer, hexamer, and larger arrays) in size from nanometers to micrometers through interlocking right- and left-hand loops (Guo, 2010; Shu et al., 2011). When its 5′/3′ helical domains of pRNA are substituted with an aptamer, siRNA, or other therapeutic molecules, the formation of pRNA-based nanostructure and their therapeutic functions is not interfered. By taking advantage of the self-assembling property of the pRNA, they have pioneered a unique approach to assemble non-covalently siRNAs targeting survivin to an anti-CD4 aptamer-fused pRNA. The resulting chimeric anti-CD4 aptamer-pRNA/pRNA-siRNA nanostructure (dimer of 25 nm in length) internalized specifically in CD4-expressing T-cells and reduced viability of CD4^+^ but not CD4^−^ T lymphocytes in culture.

#### Covalent aptamer-nanoparticle conjugates

RNA aptamers can be chemically synthesized and modified with different functional groups with relative ease, thus enabling their covalent conjugation to the nanocarriers. Recently, a 5′-NH_2_-modified PSMA aptamer was chemically synthesized and covalently decorated on the surface of a polymer composed of branched PEI grafted with PEG (PEI-PEG) for co-delivery of shRNA and chemotherapy agents (Kim et al., 2010; Figure 2D). In this design, PEG serves as a spacer to separate positively charged PEI from aptamers, avoiding the interference with the active folding of aptamers. The anthracycline class of anti-cancer drugs (e.g., doxorubicin) was physically intercalated into the double-stranded stem region of aptamers, and shRNA against the anti-apoptotic factor Bcl-xL was complexed with positive PEI polymer to form aptamer-conjugated nanoparticles. Their results demonstrated that this combinatorial formulation synergistically induced selective cell death of prostate cancer in comparison to a treatment in which the therapeutics simply are mixed.

In a similar report, Yang et al. (2012) fabricated a biodegradable nanoparticle using a poly(dl-lactic-*co*-glycolic acid) polymer that encapsulated androgen receptor (AR) shRNA molecules. After the AR shRNAs were loaded inside the particles, the surface of the nanoparticles was then conjugated with the A10 anti-PSMA aptamer for prostate cancer cell-specific targeting. A10-conjugation enhanced cellular uptake of nanoparticles in both cell culture and xenograft-based models. The efficacy of gene silencing was confirmed in PC-2/AR-derived xenografts in nude mice, and two injections of the AR shRNA-loaded A10-nanoparticles resulted in rapid tumor regressions.

In addition to siRNA/shRNA, miRNAs are another type of small regulatory RNA that silences target messenger RNAs in a sequence-specific manner MiRNAs are encoded in the genome and transcribed from endogenous miRNA genes as primary transcripts (pri-miRNAs), composed of ∼65–70 nt stem-loop structures. The mechanism of miRNA-mediated silencing is repression of target mRNA translation accompanied by deadenylation and subsequent degradation of the mRNA targets (Kurreck, 2009; Chiang et al., 2010). MiR-15a and miR-16-1 have been identified as tumor suppressor genes in prostate cancer (Bonci et al., 2008), and are associated with the expression gene that promote the survival, proliferation, and invasion in prostate cancer cells, including Bcl2, CCND1, and WNT3 A (Sherr, 1996; Dhanasekaran et al., 2001; Clevers, 2006). A truncated anti-PSMA aptamer (A10-3.2) was used as a prostate cancer cell-specific ligand for microRNA-loaded polyamidoamine (PAMAM) dendrimer delivery (Wu et al., 2011). Using a PEG as a spacer, 3′-SH modified A10-3.2 was chemically conjugated to the surface of PAMAM. The synthesized aptamer-PEG-PAMAM dendrimer effectively delivered miR-15a and miR-16-1 to prostate cancer cells overexpressing PSMA, resulting in tumoricidal efficacy. The cell viability assay showed that IC_50_ value of miRNA-loaded aptamer-PEG-PAMAM was approximately 4.7-fold lower than of miRNA-loaded non-targeting-PEG-PAMAM system, which was probably attributed to the targeting function of aptamer.

## Conclusion

In summary, RNA aptamers present a therapeutically valuable approach to treating cancer and neurological disease via direct interaction with cellular receptors or delivery of therapeutically valuable oligonucleotides. Nucleic acid-based aptamers offer properties that rival or exceed small molecules and monoclonal antibodies, including their high specificity, high affinity, relatively small size, and controlled chemical synthesis (Sanghvi, 2011). Compared to the antibody drugs, appropriate chemical modifications of therapeutic aptamers can be introduced in the solid-phase synthesis to enhance not only their bio-stability but also the pharmacodynamic (PD) and PK. In a very short span of time, nine RNA or DNA aptamers have entered the clinical development pipeline for treating a number of diseases (Ni et al., 2011a; Burnett and Rossi, 2012). For example, Pegaptanib (Macugen), a 28-mer aptamer which was chemically modified with 2′-F-Py and 2′-OMe-Pu, became the first therapeutic aptamer approved for the treatment of neovascular AMD (Ng et al., 2006). In addition, this aptamer is currently being evaluated for use in cancer treatment. Aptamers targeting cancer, described in this review, have become one of the largest categories of aptamers and will continue to do so because of the potential for aptamers to recognize cancer, but not healthy cells. Currently, a handful of aptamers against β-secretase BACE1, amyloid fibril constituent Aβ and PrP have been established for neurological disorders and are still at pre-clinical stage. In addition, various aptamers raised from a wide range of cancer targets are on their way into modern medicine.

Apart from their utility as stand-alone therapeutics, cell-type specific aptamers are readily employed as potential targeting ligands for targeted therapy. Various aptamer-mediated drug delivery systems such as aptamer-siRNA/shRNA, aptamer-enzyme, aptamer-antibody, aptamer-chemotherapy/phototherapy drugs, and aptamer-nanoparticles have been established to achieve specific target recognition, therefore improving therapeutics index as well as attenuating the overall toxicity of the drugs. The most common method used to harness the RNAi pathway for targeted gene silencing is to transfect synthetic triggers (i.e., siRNAs, DsiRNAs, or shRNAs) into cells, which is a key hurdle to the widespread use of RNAi as a therapy. Although several pre-clinical studies of aptamer-mediated siRNA targeting have been successful in the animal models of cancer and neurodegenerative diseases, siRNA activity is often compromised when it is covalently fused to an aptamer. Some design configurations only work well for a particular cell-type or molecular target. Therefore, while it is necessary to develop guiding rules for designing active conjugates, it is also important to validate the drug in a robust animal model. Additionally, the uptake kinetics of the aptamer-drugs conjugates and the mechanism how these RNAs escape from the endosome remain unclear.

Currently, few aptamers are in clinical development due to the cost and limited number of developed aptamers. RNA aptamers are innately immunogenic and unstable; however, this has been remedied by the incorporation of chemically modified nucleotides. The high cost of large-scale, high-quality long RNA aptamers with modified nucleotides limits the widespread use of aptamers as therapeutics in clinically relevant populations. Nevertheless, the advantages and future prospects of aptamers outweigh these limitations. Further developments in nucleotide synthesis technology can overcome the high cost synthesis. Moreover, it is estimated that the global market value for developing nucleic acid aptamer therapeutics will continue growing in the coming years as pharmaceutical industries are expected to invest in aptamer development (Kang and Lee, 2012). Therefore, with continued advances in SELEX technology and aptamer-based therapeutics, new aptamers will be developed for the treatment and diagnoses of cancer and neurodegenerative diseases. By incorporating nanotechnology, aptamers, and other therapeutics (siRNA/shRNA, chemotherapy agents, etc.) can be assembled in one nanocarrier, thus providing a multifunctional therapeutic nanoscale device. For these reasons, we anticipate that aptamers offer a promising new class of medicine in the near future.

## Conflict of Interest Statement

John J. Rossi is a cofounder of Dicerna Pharmaceuticals and Calando Pharmaceuticals, both are RNAi companies. All authors declare no other competing financial interests.
